# The Influence of Israel Health Insurance Law on the Negev Bedouin Population — A Survey Study

**DOI:** 10.1100/tsw.2006.06

**Published:** 2006-01-24

**Authors:** Mohammed Morad, Shifra Shvarts, Joav Merrick, Jeffrey Borkan

**Affiliations:** ^1^ Clalit Health Service, Beer-Sheva, Israel; ^2^ Division of Community Health, Beer-Sheva, Israel; ^3^ Center for Multidisciplinary Research in Aging, Beer-Sheva, Israel; ^4^ National Institute of Child Health and Human Development, Beer-Sheva, Israel; ^5^ Center of Health Policy in the Negev, Beer-Sheva, Israel; ^6^ Division of Pediatrics, Faculty of Health Sciences, Ben Gurion University of the Negev, Beer-Sheva, Israel; ^7^ Office of the Medical Director, Division for Mental Retardation, Ministry of Social Affairs, Jerusalem, Israel; ^8^ Department of Family Medicine, Brown University, Providence, RI, USA

**Keywords:** Bedouin, Negev, health services, universal health insurance, public health, Israel

## Abstract

The extension of universal health service insurance to national populations is a relatively new phenomenon. Since 1995, the Israeli National Health Insurance Law (NHIL) has provided universal health services to every resident, but the effect of this law on health and health services among minorities has not been examined sufficiently. The goals of this study were to track some of the first changes engendered by the NHIL among the Negev Bedouin Arabs to examine the effects of universal health care services. Methods included analysis of historical and health policy documents, three field appraisals of health care services (1994, 1995, 1999), a region-wide interview survey of Negev Bedouins (1997), and key informant interviews. For the interview survey, a sample of 515 households was chosen from different Bedouin localities representing major sedentarization stages. Results showed that prior to the NHIL, a substantial proportion of the Negev Bedouins were uninsured with limited, locally available health service. Since 1995, health services, particularly primary care clinics and health manpower, have dramatically expanded. The initial expansion appears to have been a marketing ploy, but real improvements have occurred. There was a high level of health service utilization among the Bedouins in the Negev, especially private medical services, hospitals, and night ambulatory medical services. The NHIL brought change to the structure of health services in Israel, namely the institution of a national health system based on proportional allocation of resources (based on size and age) and open competition in the provision of quality health care. The expansion of the pool of potential members engendered by the new universal coverage had profound effects on the Health Funds' attitudes towards Negev Bedouins. In addition, real consumer choice was introduced for the first time. Although all the health care needs of this rapidly growing population have yet to be met fully, the assurances under the Law and the new level of competition promise a higher level of service in the future.

## INTRODUCTION

The Israeli National Health Insurance Law (NHIL) that came into effect in 1995 provided universal health services to every resident of the State. It fixed an obligatory basket of services that residents receive unconditionally from the health maintenance organizations or "health funds". Every Israeli is entitled to register in the health fund of his or her choice without any limitation due to age, health condition, ethnicity, or socioeconomic status. Health services included in this basket were to be provided according to medical considerations, at reasonable quality, within a reasonable time, and at a reasonable distance from the residence of the patient. The Ministry of Health supervises the activity of the health funds with additional financial control by the State comptroller. Health services are funded by an obligatory tax on income, treasury funds, and additional payments.

The effects of the NHIL on health and health services among minorities, especially Israeli Arabs, has not been examined or discussed sufficiently. Multiple studies have investigated the Law's impact on the Jewish majority, but only a single research study[1] has been dedicated to Israeli Arabs. Even this study gave scanty attention to the Arab Bedouin population in the south of Israel. Such neglect is surprising since it has been widely suggested that this community of over 100,000 in the Negev Desert require special attention in terms of health planning, due to its unique demographic and health issues.

The goals of this study were to track some of the first changes among the Negev Bedouin Arabs engendered by the NHIL, particularly in the areas of *attitudes* and availability of *health services*. In addition to examining the effects of the implementation of the NHIL on the availability of health care in this sector, it scrutinized the awareness of the Law and the utilization of and satisfaction with the local health services among the Negev Bedouins.

## BACKGROUND ON THE ISRAELI BEDOUINS

The term *Bedouin* is derived from the Arabic term *badiah* meaning "desert". Bedouin Arab nomads have lived in the Negev, the desert in the south of what is now Israel, at least since the fifth century AD. After a major exodus to other countries during the Israeli War of Independence (1948–1949), the number of Negev Bedouins has increased from 12,000 in 1948 to more than 100,000 today, comprising 24% of the Beer-Sheva District at the end of 1995[2,3].

Israeli Bedouin society is in major social, economic, and cultural transition as a result of rapid semi-urbanization and sedentarization[4]. The sedentarization process, initiated by the Turkish Empire and subsequently by the British and the Israeli governments, has sought to relocate the Bedouins into planned settlements. The result has been stratified sedentarization forms, from semi-nomadic encampments mainly in the periphery, to spontaneous settlements and planned permanent urban towns and cities. The Bedouins in the Negev live in three types of settlements, as defined by the urbanization scale:

1. *Planned permanent settlements* supported by the government, which receive municipal, health, and other modern services. Residents are generally characterized by a high level of sedentarization and tend to work in salaried work, free trades, or day labor. Vestiges of nomadic life may remain, such as tents next to fixed permanent dwellings.
2. *Spontaneous settlements* in the vicinity of permanent settlements; Bedouins living on the outskirts or close to permanent settlements, where limited services such as health and education are offered. Bedouins in this group may retain aspects of tribal economy behavior, such as raising herds and nomadic agriculture, in addition to cash jobs and may live in clans or subclans.
3. *Peripheral settlement*; Bedouins geographically distant from any permanent settlement. This lifestyle is semi-nomadic with living structures including tents or shacks. This group has no access to modern services at its settlement locality and the tribal behavior is the hallmark at this stage.

Today, almost 60% of the Negev Bedouins live in planned villages and cities. The rest reside in the shantytown-like spontaneous settlements, with less than 10% continuing the traditional semi-nomadic lifestyle.

The Negev Bedouins have the lowest overall socioeconomic status of any social or ethnic group in Israel[2]. They have half the per capita income, twice as many children, and half the living space compared to the average Israeli. Rates of unemployment and welfare support are among the highest in Israel. Bedouin students complete secondary school matriculation exams at less than half the rate of their Jewish neighbors in the Negev or Israeli Arabs in general[2]. The low status of women and children, a common characteristic of Middle Eastern tribal traditions, still has a major impact on the lives of these groups. Polygamy is frequent among Bedouins in the Negev (20% of marriages), similar to the rate in the Gulf Arab countries[5].

## Health Status of Negev Bedouins

With the rapid movement of the Negev Bedouin Arabs to permanent settlements and urbanization, the noncommunicable diseases have begun to appear alongside the pre-existing infectious diseases, problems of nutrition, and environmental exposures. The rates of preventable diseases such as anemia, diarrhea, and accidents of children are higher than the average for Israelis. The population is also characterized by one of the highest birth rates in the world. The infant mortality rate, though low compared to other countries in the region, is estimated to be 1.5–2 times higher for Bedouins than for Israeli Jews[6]. The high frequency of birth defects among Bedouin children is a particularly serious problem among this group[6].

## History of Health Services for the Negev Bedouins

According to Ottoman estimates, the Bedouin population at the end of the 19th century was in the vicinity of 45,000 inhabitants. Poor health, poverty, and communicable diseases such as trachoma and tuberculosis were common[7]. Visits by physicians were sporadic and infrequent. Beer-Sheva hosted a small hospital and a clinic that served Turkish administrators and military personnel, where medical treatment could be obtained in return for a steep fee. Few Bedouins, however, could afford such medical services and most turned to the traditional healer, the darwish[8], or to other indigenous traditional healers for their health care needs. These conditions continued until the First World War.

In 1918, with the completion of the conquest of Palestine by Great Britain, British authorities began to operate public health services and Beer-Sheva became the hub of health services for the region. Services to the Bedouins remained scarce, a situation that continued well after the establishment of the State of Israel. The Israeli War of Independence (1948–1949) completely disrupted all pre-existing health services and brought most Western medical activities to a halt. After 1949, an Israeli military government was installed in the Bedouin areas under Israeli control that supplied limited health services to the local population. Services in Beer-Sheva were expanded, but transportation and access were often difficult for the Negev Bedouins. In the 1950s, the efforts of the military government were supplemented by Jewish voluntary efforts, though finding sufficient physicians, nurses, ambulances, and equipment remained complicated. Maternal and child health services were instituted in 1952, but their penetration into the Negev Bedouin sector was limited due to lack of resources, particularly in the peripheral areas. On the 1st of December 1966, the military government was abolished entirely.

In December 1961, the Clalit ("General") Health Fund of the Federation of Labor (previously named Kupat Holim Clalit, the major health organization in Israel [HMO], providing services to over 50% of the population) began to provide ambulatory health services to the Bedouins of the Negev. The Fund opened four primary care clinics in Beer-Sheva and within the Bedouin areas, supplying services to 10,000 people. In 1972, following an agreement with the Ministry of Health, all curative services for the Bedouins were transferred solely to the Clalit Health Fund and the Ministry of Health's role became focused on supervision and preventive medicine — primarily vaccination, maternal, and child health. This state of affairs continued until 1995, when the NHIL came into effect, covering all citizens in the State of Israel. At this time, the Clalit Health Fund insured approximately 73% of the Bedouins, while none of the other three health funds had any significant activity in this sector.

## METHODS OF THIS STUDY

The methods utilized in this study included an intensive review and analysis of historical and health policy documents, three field appraisals of health care services (1994, 1995, 1999), a region-wide interview survey of Negev Bedouins (1997), and key informant interviews.

### Document Review

The investigators undertook an intensive historical and health policy review and analysis of the literature regarding the health care of the Negev Bedouins during the 20$^{th}$ century. All available sources in Arabic, Hebrew, and English were appraised including archival material, government documents, and health fund materials.

### Health Care Services Appraisals

The field appraisals of health services available in the Negev Bedouin sector included photographic and written documentation of the existing health clinics and a survey of their current staff. Data collection, which began in December 1994 on the eve of the passage of the NHIL, included the names, numbers, working hours of physicians, and advertisements of services. The second survey was carried out 1 year after the Law went into effect in December 1995 and again in December 1999. Further information on insurance and utilization patterns was abstracted from primary sources.

### Interview Survey

#### *Data Collection*

Insights from a previously conducted focus group study among the Negev Bedouins were used to identify key issues and prepare a culturally appropriate questionnaire[9]. The survey instrument was pilot tested at two stages during development. Interviews were conducted in person, after informed consent was established.

Due to concerns about the issues of bias and disclosure, it was decided to utilize nonlocal Arabic-speaking interviewers rather than members of the local population or individuals associated with the regional medical system. An independent survey company (*El-Maidan*) was hired for this purpose. Their staff was in close contact with the research team at each stage of data collection with observations of training and collection methods.

#### *Sites and Subjects*

A sample of 515 Bedouin households was selected from different Bedouin localities, with proportional representation determined by the size of the settlement and the stage of sedentarization stages among this group. The research included Bedouin families in planned, permanent settlements, spontaneous settlement in the vicinity of the permanent settlements, and peripheral settlements. In each location, a number of Bedouin families were selected randomly and heads of households were interviewed until a predetermined quota was reached. This number reflected the relative percentage of the Bedouin residents in the settlement as compared to the Bedouin population in the Negev, according to 1995 census data. Heads of households were defined as men or women above the age of 18 years who were responsible for one family or household unit. Each head of household was asked about all members of the household. The interviewers arrived at the residence of the family, obtained consent, and verified the different sociodemographic variables before proceeding with the interview. The procedure had high cultural acceptability with only 2% of those approached refusing to participate.

### *Characteristics of the Survey Sample*

The characteristics of the sample are described in Table 1 (see also Graphs 1 and 2). The sample was demographically representative to the general Bedouin population in terms of age, sex, and settlement distribution, when compared to data from the Central Bureau of Statistics. Of the 515 heads of households interviewed, 49% were men and 51% were women. The average age was 30 years old.

**TABLE 1**
**Characteristics of the Sample (n = 515 Head of Households)**

| | | |
|---|---|---|
| Sex | Male | 49% |
| | Female | 51% |
| Age distribution of population | 18–65 | 95% |
| | 65+ | 5% |
| Marital status | Married | 80% |
| | Divorced | 3% |
| | Widowers | 3% |
| | Single | 13% |
| | No answer | 1 % |
| Number of wives (for men in sample) | One wife | 67% |
| | More than one wife | 25% |
| | No answer | 8% |
| Number of children | 2 or less children | 15% |
| | 3–6 children | 35% |
| | 7–10 children | 21% |
| | More than 10 children | 16% |
| | No answer | 13% |
| Education | No formal education | 37% |
| | Elementary school | 21% |
| | Part of high school | 15% |
| | Graduate high school | 16% |
| | Higher education | 11% |
| Settlement pattern | Permanent settlement | 64% |
| | Spontaneous settlement | 18% |
| | Periphery | 18% |
| House construction | Concrete building or mixed construction | 65% |
| | Shacks | 24% |
| | Tents | 10% |
| Self-definition of health | Satisfactory or better | 85% |
| | Not good | 15% |

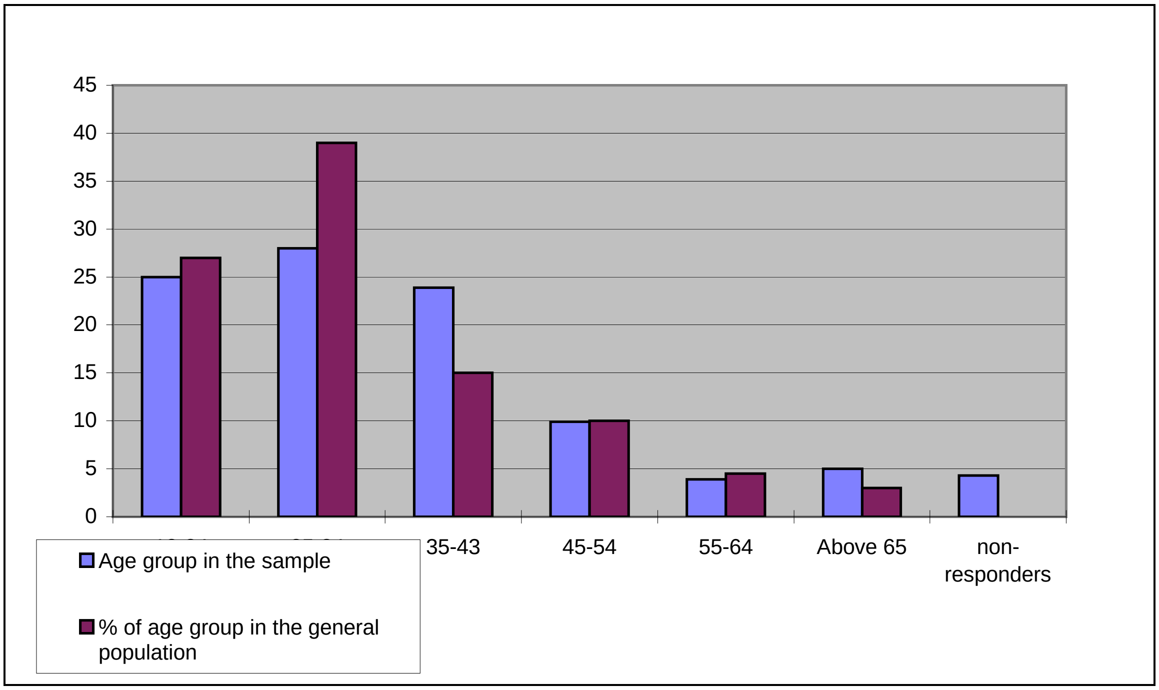

**GRAPH 1.** The distribution of the general Bedouin population above 18 years of age compared to the population of the sample in this research.

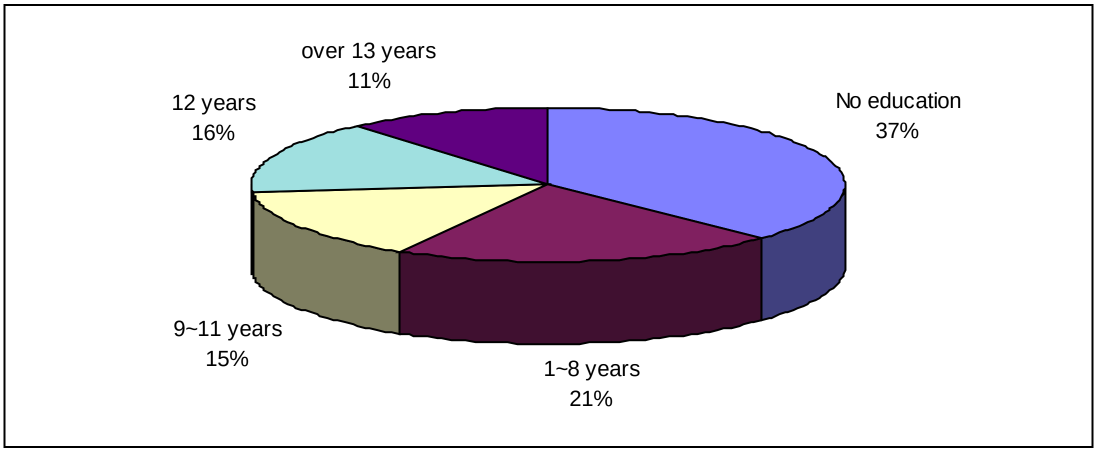

**GRAPH 2.** The distribution of the population in the sample according to the number of years of education.

## Key Informant Interviews

Key informants included health care workers in the Negev Bedouin sector, Health Fund administrators, and patients.

## Data Analysis

The survey results were analyzed using the SPSS statistical package.

## RESULTS

### Sector-Wide Effects of the NHIL on Health Care Service Among the Negev Bedouins

The enactment of the 1995 NHIL, with its provisions of universal coverage, engendered fundamental changes in health services for the Negev Bedouin sector. Under the terms of the NHIL, all Negev Bedouins received insurance coverage for the first time, a jump from the 60% rate of voluntary coverage prior to the enactment of the Law. After 1995, the national health budget was split among the four health funds proportionally, according to the number of members registered by each fund and their age group. Thus, for the first time, the Bedouin population, which had grown to encompass 100,000 souls, constituted an attractive human reservoir to bolster membership rosters. They were also seen to represent a substantial and potentially lucrative new source of net income, since they had tended to have relatively low rates of utilization of services.

Prior to 1995, the Clalit Health Fund had been the only ambulatory health provider on a large scale within the Bedouin sector. The few Bedouins who had been insured in the Meuchedet ("United") or Maccabi Health Funds before the passage of the NHIL received services in the Jewish towns and cities. As a result of the competitive environment engendered by the new law, the Meuchedet and Maccabi funds greatly increased investment in the medical infrastructure, opening competing clinics in Bedouin towns and population centers. The Clalit Health Fund also followed this trend, expanding the size and number of its branches throughout the Negev Bedouin sector. Overall, the number of health centers in Bedouin communities tripled within 2 years, with facilities scattered in every city, town, and even peripheral areas. Health care resources in this sector increased on nearly every parameter: more health funds, more clinics with wider distribution, more staff, more primary care and specialist physicians, more nurses. For example, the Clalit Health Fund increased the numbers of nurses and primary care physicians it employed in this sector by 30% soon after the enactment of the NHIL, with larger expansions by 1999 (see Table 2).

**TABLE 2**
**Trends in the Growth of Health Services (Physicians and Clinics) in Negev Bedouin Villages for the Years 1994, 1995, and 1999 by Health Funds**

| | 1994 | | | 1995 | | | 1999 | | |
|---|---|---|---|---|---|---|---|---|---|
| Fund | PCPs | Specialists* | Clinics | PCPs | Specialists* | Clinics | PCPs | Specialists* | Clinics |
| Clalit<br>% growth | 22 | 17 | 9 | 30<br>(36%) | 28<br>(64%) | 9 | 59<br>(96%) | 62<br>(121%) | 17<br>(88%) |
| Macabbi<br>% growth | 4 | 6 | 3 | 9<br>(110%) | 6 | 4<br>(25%) | 9 | 11<br>(83%) | 4 |
| Meuchedet | 2 | 2 | 1 | 2 | 2 | 1 | — | — | — |
| Leumit | — | — | — | — | — | — | 4 | 5 | 2 |
| Bedouin population | | 87,400 | | | 92,900 (6%) | | | 120,000 (29%) | |

*All specialists work part time.

PCPs = Primary care physicians.

Not all the expansion was positive, however. Scores of opportunists entered the "feeding frenzy" in the Bedouin sector after the passage of the NHIL. This included local physicians, most of them general practitioners with no residency training in primary care/family medicine, who saw an opportunity to earn money by opening clinics running in aftertime hours or businessmen who tried to engage physicians to work for them in semi-private clinics during their "off hours". These clinics were set up in rented apartments with minimal workforces, equipment, and extremely limited hours. One ploy was to name a famous or well-known physician as the head of the clinic in order to attract patients, while in reality less-qualified, cheaper physicians provided the medical services. Clinics at the scattered spontaneous settlements were closed on Fridays and most afternoons; times the population preferred.

Some of the health care service expansion was short lived. One view heard in the Negev was that much of the initial expansion in clinics and health manpower after 1995 was only a cynical marketing ploy for attracting patients to various health funds right after the implementation of the Law. There is some basis for this theory, since some of the clinics opened in 1995 by one of the health funds (Meuchedet) were closed soon thereafter, as were some offered services, such as the Maccabi scheme for mini-bus transportation to their clinics in a few of the major Bedouin towns. In addition, many of the clinics that were originally opened in 1995 by the Clalit and Maccabi Funds were located in temporary buildings, rented apartments, or even isolated rooms with little thought for long-term planning or the appropriateness of facilities for the population. Nonetheless, the overall increase in health care services in the Negev Bedouin sector that accompanied the implementation of the Law was not only maintained, but grew by 1999. The Clalit Health Fund expanded from 9 to 17 clinics between 1995 and 1999, and the Leumit ("National") Health Fund, which had had no representation in the Bedouin sector prior to the NHIL, entered the fray in 1998, opening two local clinics.

## Effects of the NHIL on the Individual: Consumer Choice and Transferring Between Funds

With the changes in the Law, Negev Bedouins were suddenly presented with universal health insurance coverage and with actual consumer *freedom of choice* for the first time. Their rights to health care could be realized at any of the four health funds, often with a variety of locally available primary care and specialist providers. The only area where access remained a significant issue appears to be in the periphery, where transportation issues, particularly in the rainy season, remained daunting. Initially four part-time clinics were set up in such areas, but two of these were closed by 1999.

Significant local turmoil, grand advertisement campaigns, and allegations of bribery to local community and tribal leaders accompanied the initial scramble for new subscribers to the health funds after the introduction of universal health insurance in 1995. Opportunists, whether agents or employees of the health funds, were paid to attract new subscribers and operated vigorously, and sometimes unethically, among the Negev Bedouins.

Parallel to the health insurance management groups' activities in recruiting new members, the Bedouins themselves quickly began to understand the advantages underlying the health insurance act, based as it was on population numbers. Heads of tribes or subtribes, who wanted to promote themselves and their status, turned directly to the health funds' management, promising to transfer their entire tribes of hundreds or even thousands of people to a specific health fund, in return for monetary or service bonuses for themselves or their families. In many cases, the health funds' management succumbed to these demands and opened mobile clinics within the residential domain of specific tribes. In other cases, which were never formally documented, under-the-table moneys are assumed to have been paid to tribal heads in return for registering their people in a health fund. Some tribal heads even increased their activity and transferred their tribes each year (as permitted by law) from health fund to health fund, while they raised their bonus prices with each transferal.

Transfers between health funds by existing members were common; 17% of the heads of households in our sample took advantage of their privilege to change a health fund membership between 1995–1997 when the general transfer rate among the rest of the population was around 4%. Most of the transfers were from the Clalit Health Fund to the Meuchedet and Maccabi Health Funds. In the 1997 survey, the Clalit Health Fund insured 73.8% of the subjects, while the Meuchedet Health Fund, which had no representation in this sector prior to 1995, had jumped to 7%. In comparison, 6.4% were insured by the Maccabi Health Fund in 1997, compared to 5.4% prior to the Law's passage.

The most common reasons given for transferring between funds were related to the range and quality of services provided, the level of the staff, and their attentiveness to their Bedouin clientele. Complaints regarding the long waiting periods and the long lines were reported by 38.7% of the Bedouins who changed a health fund. Other reasons for transfer were due to transportation difficulties (18.3%), unsatisfactory hygienic conditions in the clinic (14%), the lack of attention from the staff (5.4%), or problems with the medical staff (7.5%). Only 1.1% of the transferring Bedouins defined language as the cause to change a health fund.

## Awareness and Attitudes Regarding the NHIL

The Bedouins who participated in the sample were asked several questions regarding their rights to health care and health insurance, the implementation of these rights, and whether there had been changes in their health condition after the implementation of the NHIL.

In general, there was widespread knowledge of the NHIL, with only 3.7% of respondents reporting that they had not heard of the Law. The actual details of the NHIL were less clear to interviewees, however. Only 24% of the heads of households answered that all the citizens in the country are insured under the law and only 7% knew that individual payments for the National Health Tax are deducted from their paychecks.

Knowledge of rights under the Law was also vague. Only 5% were sure that the Law ensured treatment in any case, though 42% were aware of citizens' right to receive medical treatment with no connection to the tax payment; 54% of the Bedouin heads of households thought that the NHIL covers all health services, while 30.9% thought that it covers only part of them, 15% of those interviewed did not know what the law covers. Fully 40% did not know the procedure of transferring health fund membership.

The Negev Bedouins in the survey viewed the NHIL positively; 5% of the Bedouin families believed that the Law was favorable for all the citizens in Israel, while 4% felt that the Law primarily served the economically weak sectors of the society. The effects of the NHIL were also felt to be positive by most heads of households; 63% indicated an improvement in medical services provided to the Bedouins after the passage of the Law in 1995, while 25% thought that the standard of medical services given to the Bedouins had not changed, and 11% believed that the standard of medical services had actually worsened.

## Utilization of Health Services

The survey of heads of households provided a "snap-shot" of the health care utilization, access, and satisfaction issues among the Negev Bedouins during January–February 1997. As seen in Table 3, the results indicate a high level of health service utilization among the Bedouins in the Negev, especially for private care and hospital-based services. The vast majority (81%) of the families had a member who visited a primary care practitioner in a Health Fund clinic during the 2 months prior to the survey. Nearly one-quarter (32%) of the Bedouin families sampled used private medical services, while 44% of the families had a member who required hospital-based care. During the same 2-month period, 32% of the families in the Negev had consultations with specialists and 8% had more than three such consultations. Mother and child preventive health clinics were visited by 36% of the Bedouin families in the Negev during January–February 1997.

The use of private physicians by lower-income populations can be explained by the short clinic hours in the funds' clinics, which obliged the working population to use after-hours private services. The willingness to pay out-of-pocket money for private services among the Bedouins was similar to the willingness of the general Jewish community to do so, probably due to the importance of health issues in the culture of the two communities.

**TABLE 3**
**Number of Visits to Health Care Providers in the 2 Months Prior to the Interview by a Family Member (n = 515)**

| | Number of Visits | | | | |
|---|---|---|---|---|---|
| | **Never** | **1** | **2** | **3+** | |
| Primary care physicians | 18.8% | 12.4% | 15.1% | 53.6% | 100% |
| Specialist physicians | 67.8 | 13.6 | 10.9 | 7.8 | 100% |
| Private physicians* | 77.3 | 11.8 | 6.4 | 4.5 | 100% |
| Hospital-based clinics | 55.7 | 22.5 | 9.3 | 12.4 | 100% |
| Mother and child services | 63.7 | 19.6 | 10.1 | 6.6 | 100% |
| Traditional healers | 94.0 | 2.9 | 1.9 | 1.2 | 100% |

* Private physicians: (1) general practitioners operating their own clinics within the Bedouin settlements, (2) Health Fund physicians providing services after clinic hours in their private/home clinics, (3) Arab Palestinian physicians in the Judea, Samaria, and Gaza providing services at a lower rate.

Traditional medicine was still utilized by Negev Bedouins, although, at a much lower rate than in the past. Only 6% of the Bedouin families turned to traditional healers to receive treatment during this 2-month period. The age and sex distribution of visits were fairly evenly spread except in a few cases. Nearly half of those Bedouins who visited traditional healers (48% of 31) were women aged 18–60 years. Similarly, women aged 18–60 years dominated visits to primary care Health Fund clinics and specialists, utilizing 45% of the 418 consultations and 49% of 228 consultations, respectively.

## Access: Transportation

For the Negev Bedouins, transportation issues were central to their access to health services. There is generally no public transportation or taxi service within or between Bedouin settlements. Settlements are often located with a wide geographic distribution both between settlements and between domiciles (tents, shacks, or houses). Thus without family assistance, it is often difficult for women and children to get to the health clinics. As shown in Table 4, most Bedouins in the survey sample went by foot to the primary care or maternal and child health clinics, while public transportation and private cars and trucks were the primary means when attending private physicians and the hospital. This likely was due to both the geographic location of services — primary care and maternal and child health are nearer to domiciles, as well as the drafting of greater family or clan resources when special consultations were required — as in the case of hospitalization or private services. In 1995, the Maccabi Health Fund attempted to provide mini-bus transportation for members of their fund to and from their health centers, but this service was discontinued after 1 year. The Clalit Health Fund attempted a different solution. They introduced telemedicine between their clinics in the Bedouin villages and a network of specialists at the regional hospital.

The differences in the access for health services between primary care and specialists was dominant. On the one hand, the high utilization of primary care bridged the lack of transportation and enabled them to get primary services located within walking distance; on the other hand, Bedouins often said that the lack of public transportation prevented them mainly from using specialists as needed, due to the limitation of the specialists' clinics and the location of most specialists' services in the city of Beer-Sheva. So the access to health services was low for specialists and private physicians and high for primary care in the public clinics.

**TABLE 4**
**Means of Transportation to Health Care Facilities Among Those Utilizing the Services by Percent**

| | By Foot | Public Transportation | Animal Cart | Private Car/Truck | No Answer | n = |
|---|---|---|---|---|---|---|
| Primary care physicians | 53.6% | 20.3% | 0.2% | 24.2% | 1.7% | 418 |
| Specialist physicians | 19.3 | 37.3 | 0 | 41.0 | 2.4 | 166 |
| Private physicians | 7.7 | 47.0 | 0.9 | 42.7 | 1.7 | 117 |
| Hospital-based clinics | 1.8 | 50.0 | 0.4 | 46.9 | 0.9 | 228 |
| Mother and child services | 51.9 | 25.1 | 0.5 | 19.3 | 3.2 | 187 |
| Traditional healers | 25.8 | 32.3 | 3.2 | 32.3 | 6.5 | 31 |

### Satisfaction with Health Services

The survey of heads of households demonstrated great variation in patient satisfaction between the different health services in Negev Bedouin sector. Satisfaction was particularly low for primary care services (72%), while hospitals (87.7%), private physicians (100%), specialists (93.4%), and traditional healers (74.2%) fared somewhat better.

## DISCUSSION

### The Effects of Universal Health Insurance Law on the Bedouin Negev Sector

The NHIL created a national health system based on proportional allocation of resources and open competition dedicated to the provision of quality health care. The expansion of the pool of potential members engendered by the universal coverage aspects of the Law had profound effects on the health funds' attitudes towards Negev Bedouins. The changes in the structure of the health services insurance mechanism in Israel has resulted most significantly in three advances for this sector:

1. Universal coverage for the large proportion of previously uninsured
2. Increased investment in the health care infrastructure and expansion of services
3. Real consumer freedom of choice to choose between funds

Overall, this has brought about significant expansion of health services for the Bedouins and, presumably, improvements in service and quality. Nonetheless, not all the health-service needs of this rapidly growing population have been met. Many researchers point out the inadequate allocation of health-related resources and manpower in both the permanently settled centers and in the unplanned encampments[4,10,11,12]. In their opinion, a tremendous gap exists between actual and desired performance (in clinics working hours, location, health education and prevention programs, transportation, health personnel, etc.). This is even truer for the small nomadic Bedouin community, which does not enjoy the benefit of services provided by municipal or civil institutions.

The frequent transfer of Bedouin members between different health funds has impeded the creation of a permanent infrastructure for care and the extended medical services have achieved only part of their goal. Through examination of the movement between funds, it is evident that only 4% of Israeli Jews switched health funds within the framework of the 1995 Health Insurance Act, while the movement among the Bedouin sector has reached 7%, twice that of the Jewish sector[13].

A further problem exists, one that paradoxically is related to the universality and equality aspects of the 1995 NHIL. The movement towards uniformity in health services across ethnic groups and geographic regions in Israel was intensified with the passage of the Law. This Law guarantees an equal "basket" of health services to all citizens of the State of Israel, but provides universal coverage without any special provisos or preparations for groups with special needs. Overnight, all Bedouins became eligible for curative health care services, the details of which were unspecified in the Law. Planning for the special needs of the Bedouin minority was not done prior to the passage of the Law, nor was any surveillance or evaluation mechanisms, such as computer systems, put in place to gauge the short- or long-term consequences on this population. Even the most basic measures were not done, such as training medical staff to provide culturally compatible health services.

Such lack of consideration for the needs of minorities has been characteristic of other universal health care legislation, such as enacted in Great Britain or Australia. Presumably, considerations of equality override any particularistic needs. The general tendency in countries with national health insurance is to make services essentially the same throughout. There are exceptions, but they are limited. New Zealand, a country that, like Israel, has a relatively large indigenous population in a relatively small geographic area, maintained some special services for the Maori, but this was primarily done in 1940s with regard to special campaigns, such as combating tuberculosis. Interestingly, one of the major Western countries without national health insurance (the US) is the one where a targeted program has been created[14,15].

More limited universal health care laws have been enacted in Israel before, but they only pertained to maternal and child health preventive care service. In 1954, the Knesset (the Israeli Parliament) passed a national insurance law that mandated prenatal services for all pregnant women and well-child preventive health services for all children. This law had enormous impact among Israeli Arabs, including the Negev Bedouins. Whereas in 1954, only 5% of women in the Arab sector had hospital deliveries, this increased to 80% by 1965 and 100% today[16,17].

## Awareness of the NHIL and Its Consequences

The survey indicated that although there was wide knowledge of the existence of the NHIL, there were significant gaps regarding its content and implications. This was particularly true regarding membership rights in the health funds and the privilege of transferring between funds. This could indicate a problem in the information dissemination among the Bedouins in the Negev. Issues of literacy and language are key, such as the tendency of the health funds to publish some materials in Hebrew only.

## Satisfaction

With very few exceptions, health and health policy studies of the Negev Bedouins have paid little or no attention to their preferences, beliefs, and satisfaction[18,19]. No comprehensive study has yet been undertaken to examine the Bedouins' satisfaction with the health service. Due to the lack of prior data, comparisons are difficult. However, a study conducted in 1997 by the Brookdale Institute[1] found higher levels of satisfaction among Israeli Jews and Arabs than those seen in this survey. The study discerned no significant differences in the level of satisfaction rate with health fund services between Jews and Arabs. This study also found extensive use of private medical services in the minority sector, particularly in the hours after the health funds' clinics close. The Brookdale survey interviewed few Negev Bedouins due to the sampling method and it largely represents those Muslim, Christian Arab, and Druse who live in central and northern Israel. In our study, only 72% of Negev Bedouins were satisfied with health fund services, while the Brookdale survey found satisfaction among Israeli Arabs and Jews for health funds of 89% and 83%, respectively[1].

The Brookdale Institute survey also found that Israeli Jews and Arabs were less convinced that health-service provision had improved after the implementation of the NHIL than the Negev Bedouins who were part of our survey. Israeli Jews and Arabs were also not as sure that this Law had had a positive effect on the equality of treatment as compared to the Negev Bedouin Arabs. Such results may reflect two changes:

(1) actual improvement of health services and (2) differences in expectations regarding future improvements in services.

Overall, the NHIL must be viewed as an important step towards the advancement of health services in the Bedouin Arab sector and toward the reduction in the real gaps in services between Israeli Jewish and Arab populations. However, the improvements in the Negev Bedouin sector may have been the largest, since correspondingly, the room for improvement was greatest there. Not only were more Bedouins uninsured than any other sector, but health facilities were also the most geographically distant from their target group.

### Utilization of Services

Among the Bedouin population in the Negev, there was intense utilization of hospital services (45%) and primary services (81%) for every person in the family during the 2 months prior to sampling. Women aged 18–60 represent the largest percentage of health services users. This figure could be explained by the Bedouin woman's low social status and sick behavior that characterizes such status.

The use of traditional medical service is decreasing (6%) and could indicate the reduction of the role of this service among the Bedouins in the Negev. This may be due to a combination of the effects of modernization, increased sedentarization, and greater access to Western-type medical care.

### Ethical Considerations

Some of these findings raise fundamental issues regarding ethical dimensions of the passage of universal coverage. Were the interests of the minority safeguarded under the NHIL? Is a minority, particularly one with little experience in the health care marketplace, really equipped to make "mature and educated" decisions when many choices are presented through the filter of modern marketing camouflage? At what stage should the minority be involved in making wide-range health policy changes and what preparation is needed prior to allowing choice?

## RECOMMENDATIONS

The universal health care that was heralded in by the NHIL has had significant, positive impact on the health services for individuals in this sector. Nonetheless, this community deserves the attention of researchers and health planners because of the demographic and health problems that continue to contribute to higher morbidity and excess neonatal mortality, compared with the Jewish and Northern Israeli Arab community. In order to improve the health condition of the Bedouin population in the Negev, health policy planners must consider the impact of the NHIL on this sector and must tailor health services to the special needs and the dynamic social transition of this population. For example, a positive first step would be to encourage the Health Ministry to adjust the distribution of health services in the Bedouin sector in the Negev to the geographic, demographic, and cultural realities specifically present in the Negev.

Finally, there is a need to form integrated multidisciplinary research teams including medical, management, social science, and economic experts, in order to study the effect of the urbanization process on the Bedouins' health conditions well as the compatibility of the medical services with the Bedouin population's cultural needs.

## ACKNOWLEDGMENTS

The authors are deeply grateful for the advice, the valuable criticism, and the special help of Prof. Stephen J. Kunitz, Community and Preventive Medicine, Division of Social and Behavioral Medicine, University of Rochester Medical Center. This research was funded by grants from the Israeli Institute for National Health Policy and Health Services Research and by the Israel Ministry of Health Research Fund.

## BIOSKETCHES

**Mohammed Morad, MD**, is specialist in family medicine; lecturer in family medicine affiliated with the National Institute of Child Health and Human Development, Division of Community Health and Center for Multidisciplinary Research in Aging, Faculty of Health Sciences, Ben Gurion University of the Negev; and the medical director of a large area clinic in the city of Beer-Sheva operated by the Clalit Health Services. Dr. Morad's publications include those on Bedouin health, health aspects, spiritual health, and aging in persons with intellectual disability, and he is a presenter at national and international conferences on topics including health policy and services for the disadvantaged. E-mail: morad62@013.net.il

**Shifra Shvarts, PhD**, is professor and director of the Center of Health Policy, Faculty of Health Sciences, Ben Gurion University, Beer-Sheva, Israel. With a BA in history and geography, an MA in history, and a PhD in health sciences, her interests and numerous publications center around the history of medicine and public health, development of the health care system in Israel, development of health organizations, health insurance legislation, and the development and history of maternal and infant welfare centers. E-mail: shvarts@bgumail.bgu.ac.il; Website: http://fohs.bgu.ac.il/people/PDetails.asp?StaffID=11733

**Joav Merrick, MD, DMSc,** is professor of child health and human development affiliated with the Center for Multidisciplinary Research in Aging, Zusman Child Development Center, Division of Pediatrics and Community Health at the Ben Gurion University, Beer-Sheva, Israel; the medical director of the Division for Mental Retardation, Ministry of Social Affairs, Jerusalem; and the founder and director of the National Institute of Child Health and Human Development. Dr. Merrick has numerous publications in the field of child health and human development, rehabilitation, intellectual disability, disability, health, welfare, abuse, advocacy, quality of life and prevention and he received the Peter Sabroe Child Award for outstanding work on behalf of Danish Children in 1985 and the International LEGO-Prize ("The Children's Nobel Prize") for an extraordinary contribution towards improvement in child welfare and well being in 1987. E-mail: jmerrick@internet-zahav.net; Website: www.nichd-israel.com

**Jeffrey Borkan, MD, PhD,** is professor and chairman of Family Medicine, Brown University, Providence, RI, since August 2001. He is a family physician educator, researcher, clinician, and administrator whose career has spanned two fields (family medicine and medical anthropology) and two countries (the US and Israel). He came to Memorial Hospital of Rhode Island and Brown Medical School after having been the Vice Chair of Behavioral Science at Tel Aviv University, Sackler Faculty of Medicine. Throughout his career, he has been active in family medicine research, as well as being a practicing family physician and resident/student trainer. Research includes the physician-patient relationship, health policy, low back pain, and storytelling in medicine. He has frequently worked and conducted research among minorities and the underserved, both in the US, Israel, and the Pacific Island of Tonga. E-mail: Jeffrey_Borkan@mhri.org
Website:www.brown.edu/Divisions/Medical_School/andera/person.php?id=2095&returnURL=%2FDivisions%2FMedical_School%2Fandera%2Ffaclist.php%3Ffirst%3DB
